# Effects of Light and Temperature on the Metabolic Profiling of Two Habitat-Dependent Bloom-Forming Cyanobacteria

**DOI:** 10.3390/metabo12050406

**Published:** 2022-04-29

**Authors:** Bijayalaxmi Mohanty, Seyed Mohammad Majedi, Shruti Pavagadhi, Shu Harn Te, Chek Yin Boo, Karina Yew-Hoong Gin, Sanjay Swarup

**Affiliations:** 1 NUS Environmental Research Institute, National University of Singapore, Singapore 117411, Singapore; eriv97@nus.edu.sg (B.M.); majedi@nus.edu.sg (S.M.M.); pavagadhis@nus.edu.sg (S.P.); eritsh@nus.edu.sg (S.H.T.); chekyin220@hotmail.com (C.Y.B.); ceeginyh@nus.edu.sg (K.Y.-H.G.); 2 Singapore Centre for Environmental Life Sciences Engineering, National University of Singapore, Singapore 117456, Singapore; 3 Department of Civil and Environmental Engineering, National University of Singapore, Singapore 117576, Singapore; 4 Department of Biological Sciences, National University of Singapore, Singapore 117558, Singapore

**Keywords:** cyanobacteria, *Hapalosiphon*, *Planktothricoides*, light, temperature, metabolomics

## Abstract

Rapid proliferation of cyanobacteria in both benthic and suspended (planktonic) habitats is a major threat to environmental safety, as they produce nuisance compounds such as cytotoxins and off-flavors, which degrade the safety and quality of water supplies. Temperature and light irradiance are two of the key factors in regulating the occurrence of algal blooms and production of major off-flavors. However, the role of these factors in regulating the growth and metabolism is poorly explored for both benthic and planktonic cyanobacteria. To fill this gap, we studied the effects of light and temperature on the growth and metabolic profiling of both benthic (*Hapalosiphon* sp. MRB220) and planktonic (*Planktothricoides* sp. SR001) environmental species collected from a freshwater reservoir in Singapore. Moreover, this study is the first report on the metabolic profiling of cyanobacteria belonging to two different habitats in response to altered environmental conditions. The highest growth rate of both species was observed at the highest light intensity (100 μmol photons/m²/s) and at a temperature of 33 °C. Systematic metabolite profiling analysis suggested that temperature had a more profound effect on metabolome of the *Hapalosiphon*, whereas light had a greater effect in the case of *Planktothricoides*. Interestingly, *Planktothricoides* sp. SR001 showed a specialized adaptation mechanism via biosynthesis of arginine, and metabolism of cysteine and methionine to survive and withstand higher temperatures of 38 °C and higher. Hence, the mode of strategies for coping with different light and temperature conditions was correlated with the growth and alteration in metabolic activities for physiological and ecological adaptations in both species. In addition, we putatively identified a number of unique metabolites with a broad range of antimicrobial activities in both species in response to both light and temperature. These metabolites could play a role in the dominant behavior of these species in suppressing competition during bloom formation. Overall, this study elucidated novel insights into the effects of environmental factors on the growth, metabolism, and adaptation strategies of cyanobacteria from two different habitats, and could be useful in controlling their harmful effects on human health and environmental concerns.

## 1. Introduction

Cyanobacterial blooms in water bodies are an increasing worldwide threat to both human and aquatic organisms. Some of the cyanobacterial species multiply rapidly and form benthic or planktonic blooms due to an increase in unfavorable environmental conditions, such as water eutrophication due to urbanization and agriculture, global warming [1,2,3,4], and water pollution by herbicides [5] and industrial waste. These blooms have severe impacts on the ecosystem, such as changes in biodiversity, light, and oxygen concentration and the habitat of aquatic organisms. These benthic or planktonic blooms can produce a wide range of noxious secondary metabolites, including potent toxins and odors [6] such as microcystins (MCs) and cylindrospermopsin [7,8], which can influence terrestrial and aquatic organisms [9], as well as human health. Hence, it is essential to restrict the risks of cyanotoxins, as well as the production of off-flavor compounds such as geosmin and 2-methylisoborneol (2-MIB), to reduce these incidents’ occurrence due to both planktonic and benthic cyanobacteria.

The off-flavor compounds produced by both planktonic or mats of benthic type cyanobacteria are a widespread odor problem in drinking water and fish production worldwide [10,11]. The muddy or earthy–musty odor in the water usually occurs due to the secretion of volatile metabolites such as geosmin and 2-MIB. Both 2-MIB and geosmin are terpenoids, and are synthetized by terpene synthases [12]. Temperature and light irradiance are the key factors in regulating the occurrence of algal blooms and production of both 2-MIB and geosmin through various upstream primary metabolic processes [11,13]. Genes involved in the biosynthesis of 2-MIB were found in cyanobacteria in [14], and the authors suggested that the transcription of 2-MIB synthesis genes was light-regulated. The production of the off-flavor compound 2-MIB in response to different light intensities and temperature has been experimentally verified in a free-living filamentous planktonic cyanobacterium*—Planktothricoides* sp. SR001—obtained from a freshwater reservoir in Singapore [14]. They also identified the genes associated with the production of this compound. Similarly, the benthic cyanobacterium *Hapalosiphon* sp. strain MRB 220 isolated from a benthic cyanobacterial mat gathered from a sediment sample of an urban freshwater water body in Singapore also demonstrated the capability of nitrogen fixation and production of the off-flavor compounds 2-MIB and geosmin [15].

Temperature is one of the most important factors in the growth and metabolism of cyanobacteria in the absence of nutrient shortage [16], as most of the metabolic reactions are temperature-dependent. It has been shown that cyanobacteria outcompeted other algae at elevated temperature, and their toxin content also increased with higher growth rates [17,18,19]. Changes in temperature often mediate production of secondary metabolites that are coupled with photosynthesis [20,21]. In addition, the higher production of these metabolites is also coupled with the elevated temperature, which is optimal for the growth of the cyanobacteria [22]. Similarly, light plays a key role in the growth and pigment accumulation of cyanobacteria. Changes in light irradiance significantly affect pigment composition that is linked to light-harvesting mechanisms [23,24]. These changes in pigmentation are mainly due to the variations of red-colored phycoerythrin and blue-colored phycocyanin [25], and any slight change in the light-harvesting mechanism may lead to changes in the morphology and physiology of cyanobacteria. Nevertheless, the effects of environmental factors on the growth and metabolic pathways of algal blooms are not fully understood yet.

To date, the majority of the studies on cyanobacterial blooms have focused on the model organism *Microcystis* and the bloom-producing planktonic forms of cyanobacteria that are associated with toxicity, which are risky for the aquatic ecosystem [26,27]. However, studies on benthic cyanobacteria that produce the common toxin microcystin have been gaining attention in the scientific community [28,29,30]. Benthic and planktonic cyanobacteria are ecologically distinct organisms that bloom in different habitats and have different evolutionary histories, which could be associated with their specific metabolic machineries. *Hapalosiphon* sp. is a nitrogen-fixing filamentous cyanobacteria, whereas *Planktothricoides* sp. is a branched cyanobacteria without the ability to fix atmospheric nitrogen. The mode of survival strategies, off-flavor production, and the impact of environmental factors on the production of microcystin and their metabolic networks are not well understood yet. Hence, we aimed to explore the impact of environmental factors such as light and temperature on the growth behavior and primary metabolic networks of both *Hapalosiphon* sp. MRB220, a benthic strain isolated from the benthos in Singapore, and *Planktothricoides* sp. SR001, a planktonic strain isolated from a freshwater reservoir in Singapore.

A number of studies so far were conducted mainly to characterize different toxin-producing cyanobacteria [31,32]. However, the study of the effects of environmental conditions that promote the proliferation of both benthic and planktonic freshwater cyanobacteria is very limited. The recent advancement in omics approaches provides insights associated with the physiological and biochemical characteristic linked to the proliferation of these cyanobacteria. Environmental metabolomics approaches have been utilized to assess the organism–environment interactions at the molecular level to improve the understanding of their mode of action [33,34]. Through this application, identification of different metabolites assists in detecting alterations in certain metabolic pathways under environmental stresses [35]. It also helps to identify the cellular activity of the organism in response to different contaminant-related stressors. Several metabolomics technologies have been developed to improve the measurement processes for specific groups, classes, and individual metabolites. This metabolomics approach has been employed to analyze different metabolic networks in cyanobacteria, such as inorganic carbon acclimation, glycogen biosynthesis processes, and structural diversity of metabolites [36,37,38]. Therefore, we aimed to utilize the metabolomics approach to elucidate the effects of environmental factors on the cellular/metabolic processes of both *Hapalosiphon* sp. and *Planktothricoides* sp. This will assist in improving our understanding of the mode of action and proliferation of these two cyanobacteria in response to light and temperature, which have structural and behavioral differences. Additionally, it may provide information on the production of multiple biofuels and bioproducts for biotechnology application, as well as the development of models for management of eutrophication problems.

## 2. Results

### 2.1. Effects of Different Light Intensities and Temperatures on the Rate of Growth of Hapalosiphon sp. and Planktothricoides sp.

We observed that the growth rates of both *Hapalosiphon* sp. and *Planktothricoides* sp. were strongly dependent on the light intensity. The increase in the light intensity from 25% to 50% and to 100% increased the growth rates. The highest growth rate (0.08 day^−1^) in *Hapalosiphon* sp. was observed at the highest light intensity (100 ± 3 μmol photons m^−2^s^−1^) (Table 1). However, the growth rate was much slower under a low light intensity (10 ±3 μmol photons m^−2^s^−1^) due to insufficient light energy to carry out oxygenic photosynthesis. This light-dependent growth rate could be related to the habitat and growth pattern of *Hapalosiphon* sp., which belongs to mat-forming benthic cyanobacteria [15]. A similar growth pattern was also observed for the planktonic cyanobacterium *Planktothricoides* sp. (Table 1), and this again was related to their habitat, as they grow on the surface of water and need an ample light intensity to grow freely and aggregate.

The growth rates of both *Hapalosiphon* sp. and *Planktothricoides* sp. were found to be the highest at 33 °C (Table 2). In *Hapalosiphon* sp., it was almost similar (0.06 ± 4 day^−1^) at 25, 28, and 38 °C. However, this trend was different in *Planktothricoides* sp., which had a slight increase in growth rate as the temperature increased from 25 to 33 °C (Table 2). A further increase in temperature to 38 °C had a negative impact on the growth rate. This suggested that *Planktothricoides* sp. may not be able to withstand higher temperatures of 38 °C and higher.

### 2.2. Effects of Light and Temperature on the Metabolites of Hapalosiphon sp. and Planktothricoides sp.

Metabolomic profiling was performed to understand the effects of light and temperature on the key metabolites and respective metabolic pathways of both *Hapalosiphon* sp. and *Planktothricoides* sp. Cultures grown under four different light intensities/temperatures were harvested at four time points (0, 16, 20, and 24 days). The metabolites obtained from the samples were putatively identified using in-house (for Microcystis aeruginosa) and available databases such as METLIN (metlin.scripps.edu) (accessed on 15 November 2020). After identification, the differential metabolites were mapped to pathway networks using the reference database for model cyanobacteria (Microcystis aeruginosa in the KEGG pathway). We observed that both light and temperature conditions significantly affected the metabolic profiling of both species after 24 days of treatment.

#### 2.2.1. Effects of Light Intensity on the Metabolite Profiling of *Hapalosiphon* sp. and *Planktothricoides* sp. (Light Intensity and Time Point as Variables)

**Figure 1:**
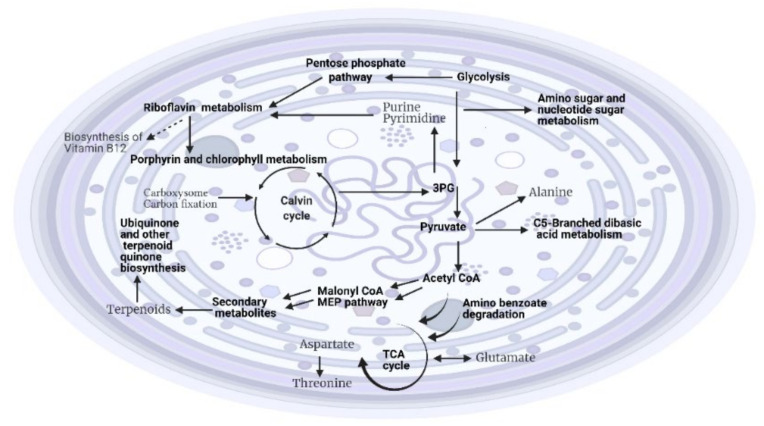
Cellular model representing the effects of light on the metabolic activities of *Hapalosiphon* sp. (MRB 220) based on metabolic profiling. The metabolites corresponding to pathways shown in the grey color were partially identified. Created with BioRender.com. (accessed on 4 August 2021).

The metabolite profiling of *Hapalosiphon* sp. grown under different light intensities identified several features. It appeared that the metabolite profiles were influenced by light (19% of variation), while time-point differences could explain 17% (see Table 3) of the total variation. The interaction effect between light and time points was significant. The residuals (unknown sources of influence) accounted for nearly 46% of the variation. Statistical analyses carried out using a principal component analysis showed an effect of light on the metabolite profile differences (Table 3). Differentially abundant mass features based on light identified 41 putative metabolites. After mapping those putative metabolites onto the KEGG metabolic pathway in *Microcystis aeruginosa*, the final number of putative metabolites was reduced to eight (Supplementary Materials S1). Among those, metabolites linked to different amino acid biosynthesis included alanine, aspartate, and glutamate metabolism, as well as arginine and proline metabolism. We also identified metabolites linked to purine and pyrimidine metabolism, riboflavin, C5-branched dibasic acid and amino sugar, nucleotide sugar metabolism, and microbial metabolism in diverse environments (Figure 1). Moreover, metabolites linked to ubiquinone and other terpenoid–quinone biosynthesis were also present. Some of these pathways were also found to be linked to precursors for downstream synthesis of secondary metabolites, such as off-flavor metabolites. Metabolites associated with riboflavin metabolism can be linked to chlorophyll and porphyrin metabolisms, which lead to the production of vitamin B12. Interestingly, our analyses also identified new pathways such as microbial metabolism in diverse environments, as well as ubiquinone and other terpenoid–quinone biosynthesis, which can be linked to amino benzoate degradation. Amino sugars and nucleotide sugars could be associated with energy production during high light intensity. The identification of metabolites belonging to ubiquinone and other terpenoid–quinone biosynthesis suggested their roles in oxidative phosphorylation and amino benzoate degradation.

**Figure 2:**
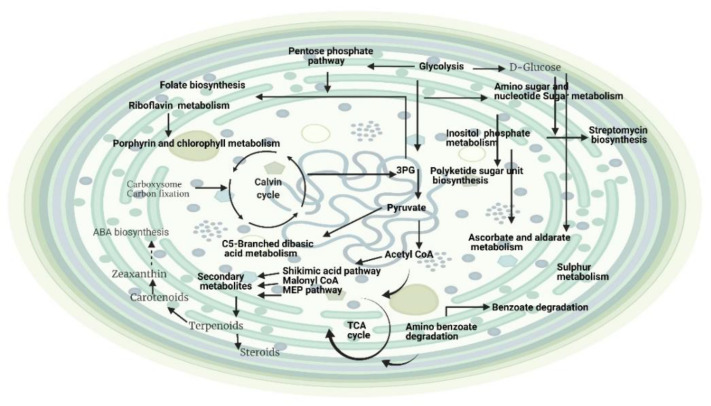
Cellular model representing the effects of light on the metabolic activities of *Planktothricoides* sp. (SR001) based on metabolic profiling. The metabolites corresponding to pathways shown in the grey color were partially identified. Created with BioRender.com (accessed on 4 August 2021).

The metabolite profiling of *Planktothricoides* sp. grown under different light intensities identified a few features. As shown in Table 4, the PerMANOVA analyses indicated that the major source of differences in the metabolite profiles were influenced by light, amounting to nearly 24% of variation, while time-point differences could explain only 7%. Interestingly, the residuals (unknown sources of influence) accounted for nearly 68% of the variation. The statistical analyses showed that different light intensities resulted in differences in the metabolite profile ( Table 4). The statistical analyses identified 75 mass features that were differentially abundant based on light, while differentially abundant mass features between time points were 33 after mapping onto the KEGG metabolic pathway in *Microcystis aeruginosa*, (Supplementary Materials S2). Different light intensities had a remarkable impact on the metabolism of *Planktothricoides* sp., and a wide range of different metabolic pathways were identified (Figure 2). Metabolic networks associated with light intensities were related to metabolism of amino acids such as alanine, aspartate, glutamate, lysine, arginine, serine, threonine, glycine, proline, D-glutamine and D-glutamate, cysteine, methionine, tryptophan, and histidine. The analyses also identified most of the core primary metabolic pathways. In addition, a number of important secondary metabolic pathways, such as microbial metabolism in diverse environments, ascorbate and aldarate metabolism, carotenoid biosynthesis, biosynthesis of terpenoids and steroids, polyketide sugar unit biosynthesis, and folate biosynthesis, were also identified. The analyses also showed that the light intensity had a tremendous impact on the biodegradation capacity of *Planktothricoides* sp., as several metabolites were linked to degradation of aromatic compounds, and degradation of benzoate, amino benzoate, chlorocyclohexane, and chlorobenzene had occurred. Interestingly, some secondary metabolic pathways associated with the biosynthesis of antimicrobial compounds such as ansamycin and streptomycin were also identified.

The results of this metabolomics approach clearly indicated that the light intensity had a critical impact on both the benthic and planktonic species of cyanobacteria, and their varied behaviors could have been due to their morphological and habitat differences. The mode of response to the light intensity also affected their specific roles in producing secondary metabolites with different important functions.

#### 2.2.2. Effects of Temperature on the Metabolite Profiling of *Hapalosiphon* sp. and *Planktothricoides* sp. (Temperature and Time Point as Variables)

**Figure 3:**
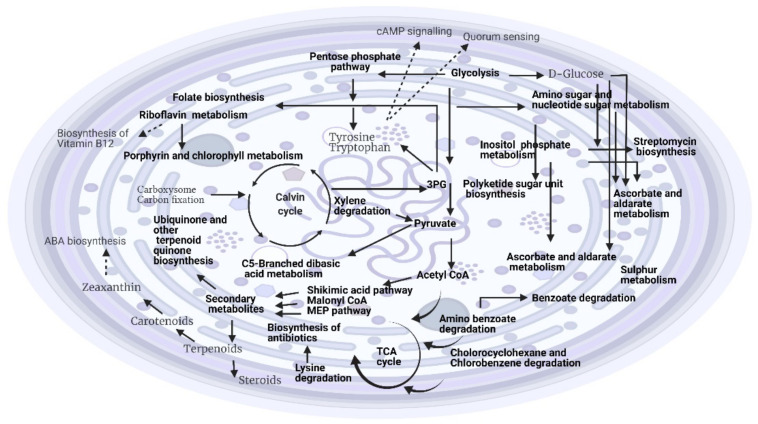
Cellular model representing the effects of temperature on the metabolic activities of *Hapalosiphon* sp. (MRB 220) based on metabolic profiling. The metabolites corresponding to pathways shown in the grey color were partially identified. Created with BioRender.com (accessed on 4 August 2021).

Several molecular features were identified for *Hapalosiphon* sp. grown at different temperatures. The metabolite profiles were influenced by growth/time point (19% of variation), while temperature differences could explain 28% (see Table 5). The interaction between temperature and time point accounted for 13% of the variation, while the residuals (unknown sources of influence) accounted for nearly 40% of the variation (Table 5). Differentially abundant mass features based on temperature were mapped onto the KEGG pathway, leading to the identification of 151 putative metabolites. The final number of putative metabolites was reduced to 43 after mapping onto the KEGG metabolic pathway in *Microcystis aeruginosa* *(*Supplementary Materials S1) (Figure 3). The metabolites linked to several primary and secondary metabolic pathways were identified in response to the temperature effects. Among them, the amino acid metabolism such as alanine, aspartate and glutamate, tyrosine, lysine, phenylalanine, histidine, arginine, proline, glycine, cysteine, methionine, serine, and threonine were identified. Metabolites linked to important secondary metabolic pathways such as microbial metabolism in diverse environments, carotenoid biosynthesis, ubiquinone and other terpenoid–quinone biosynthesis, polyketide sugar unit biosynthesis, folate biosynthesis, monobactam biosynthesis, biotin metabolism, and sphingolipid metabolism were also identified. In response to temperature, *Hapalosiphon* sp. had the ability to biodegrade a number of compounds such as chlorocyclohexane and chlorobenzene degradation, benzoate, xylene and ethylbenzene, as well as degradation of aromatic compounds. The results also revealed an interesting metabolite; i.e., L-adrenaline, which is linked to quorum sensing. These molecules may act as inhibitors of bacterial quorum sensing. However, their mechanism of inhibition is not clear [39].

**Figure 4:**
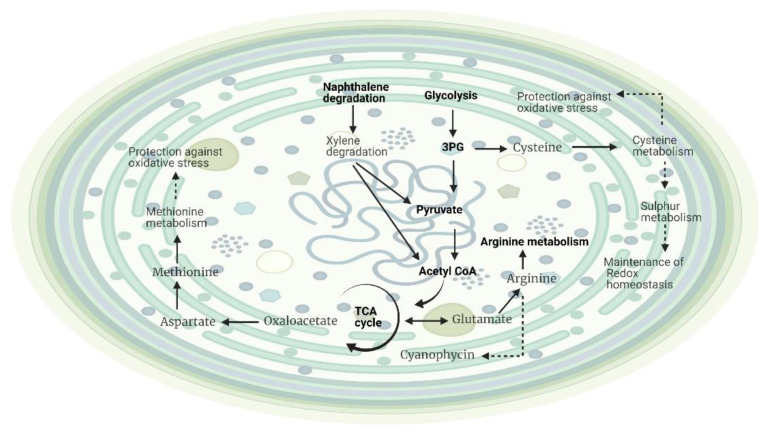
Cellular model representing the effects of temperature on the metabolic activities of *Planktothricoides* sp. (SR001) based on metabolic profiling. The metabolites corresponding to pathways shown in the grey color were partially identified. Created with BioRender.com (accessed on 4 August 2021).

The effects of different temperatures on the metabolic profiling of *Planktothricoides* sp. identified a few molecular features. As reported in Table 6, the differences in the metabolite profiles were influenced by growth/time point (30% of variation), while temperature differences could explain only 7%. The interactions between temperature and time points were also significant. Interestingly, the residuals accounted for nearly 50% of the variation (Table 6). Similarly, 107 differentially abundant mass features based on temperature were mapped onto the KEGG pathway (*Microcystis aeruginosa)*, leading to the identification of five putative metabolites *(*Supplementary Materials S2) (Figure 4). The metabolic pathways linked to the identified metabolites were surprisingly very few in response to the temperature effect in this planktonic species. They included arginine biosynthesis, cysteine and methionine metabolism, microbial metabolism in a diverse environment, and monobactam biosynthesis. However, this species still maintained the capability to degrade naphthalene and aromatic amino acids.

The metabolomics analyses evidently indicated that temperature had a significant impact on both the benthic and planktonic species of cyanobacteria and that they behaved differently, which could have been due to their structural and habitat differences. The mode of response to the temperature also affected their specific roles in producing secondary metabolites with different important functions.

## 3. Discussion

Alterations in environmental variables such as temperature, light intensity, partial pressure of water, high nutrients, or low turbidity could trigger an increase in the cyanobacterial bloom in both frequency and intensity, subsequently affecting metabolic activities for the production and release of algal off-flavors/toxins/pharmaceutical compounds [40,41]. The metabolic profiling of both species studied here was important, as it provided essential metabolism information linked to their growth and compound biosynthesis.

### 3.1. Effects of Different Intensities of Light on the Growth and Metabolic Profiling of Hapalosiphon sp. MBR220 and Planktothricoides sp. SR001

Our findings indicated that a relatively high light intensity (100 ± 3 μmol photons m^−2^s^−1^) was necessary for the maximum growth of *Hapalosiphon* sp. (Table 1). The effect of light intensity on the growth rate of other free-floating cyanobacteria such as *Planktothrix* sp. showed that a light intensity of 85 μmol photons m^−2^ s^−1^ was optimum for growth in laboratory conditions [42], whereas for the benthic *Hapalosiphon* sp*.,* the optimum light requirement was observed at 100 ± 3 μmol photons m^−2^s^−1^ in laboratory conditions, which was slightly higher than the requirement for *Planktothrix* sp. The growth of *Planktothricoides* sp. was also maximized at a high light intensity (100 ± 3 μmol photons m^−2^s^−1^), which could be correlated with their planktonic habitat, as they normally bloom at the surface of water where light intensities are stronger (Table 1).

The metabolic profiling suggested that light had some impact on the biosynthesis and metabolism of different amino acids (alanine, aspartate and glutamate, glycine, serine, threonine, arginine, and proline), leading to higher syntheses of proteins, nucleic acids, and regulatory molecules (Figure 1). These amino acids play many roles in the defense system, such as osmotic regulators, free radical scavengers, and stress signal molecules [43,44,45]. Although it has been reported that environmental conditions affected the content of amino acids in cyanobacteria, the effects of light and temperature on amino acid content has not been sufficiently studied [46,47]. Glutamate plays an important role in nitrogen metabolism [48], chlorophyll biosynthesis [49], and the adaptation of cyanobacteria to higher light intensities. Asparagine can reabsorb the released free amino acids to reduce the toxic effect of ammonia [50,51]. Serine, threonine, and tyrosine might be involved in the metabolic reactions associated with photosynthesis [52]. The intensity and quality of light also activate various signal transduction pathways that regulate physiological adaptation [53]. The identification of riboflavin metabolism can be linked to the growth of *Hapalosiphon* sp., as it is linked to chlorophyll and porphyrin metabolism through the vitamin B12 pathway. Benthic cyanobacteria could produce a large amount of vitamin B complex and vitamin E [54], and release the excess quantity to their surrounding environment. It has been reported that nitrogen-fixing cyanobacteria excreted more vitamin B12 compared to non-nitrogen-fixers [55]. The identification of the riboflavin pathway also suggested that this species of benthic cyanobacteria can be used as a valuable source of vitamins for commercial purposes [56]. Our results showed that under high light conditions, *Hapalosiphon* sp. grew through primary metabolic pathways as well as with environmental pollutants as a growth substrate, which showed their roles in the degradation of industrial pollutants and in cleaning up contaminated environments. The presence of C5-branched dibasic acid metabolism could be associated with energy production during high light intensity. Overall, the effects of different light intensities on the metabolites of *Hapalosiphon* sp. suggested that primary energy-producing metabolic pathways and amino acid metabolism are the main metabolic pathways that could have an impact on their growth and survival, which was related to their benthic habitat (Figure 1). Surprisingly, we did not identify any metabolite directly associated with secondary metabolic pathways.

Light intensity showed a greater effect on the metabolic profiling *of Planktothricoides* sp. compared to that of benthic *Hapalosiphon* sp., as higher number of different amino acid biosynthesis processes and metabolites associated with different primary and secondary metabolic pathways were identified (Figure 2). A higher number of secondary metabolites showed an enhancement of different secondary metabolic pathways in this species in response to different light intensities. Since these secondary metabolites are not vital for the survival of *Hapalosiphon* sp., these could be produced in response to high light intensities. The function involved could be for photoprotection, biodegradation, antioxidant activity, and defense against the response to abiotic and biotic stresses [57]. Carotenoids provide photoprotection of the intracellular molecules in cyanobacteria [58,59]. However, the content of carotenoids is strain-specific and depends on different environmental/culture conditions, particularly light intensity [60,61]. In addition, accumulation of b-carotene could lead to the biosynthesis of abscisic acid (ABA) to regulate the stress-response mechanism during abiotic stress responses (Figure 2). Hence, microalgal ABA signaling may share some functions with higher plants, particularly in the stress-response mechanism. It was reported that extracellular ABA was produced in response to salt stress by the cyanobacteria *Nostocl nuscorum, Trichormus uariabilisa,* and *Synech. Ococculeso poliens* in a culture medium [62]. In addition, these cyanobacteria producing different terpenoids in response to different light intensities could be beneficial for synthetic production of pharmaceutical and industrial compounds. For instance, *Hapalosiphon* sp. has the ability to biodegrade amino benzoate and benzoate under varied light conditions. Interestingly, the identification of metabolites linked to streptomycin biosynthesis suggested that they could be used synthetically as a target for antibiotics. However, the metabolic activity was still not fully functional compared to that in the growth stage, which could be due to an insufficient light intensity compared to their growth in natural habitat.

The effects of different light intensities on metabolite identification in both species, which differ in habitat and structure, clearly indicated that they responded to a change in environmental conditions according to their habitat. Higher light intensities can also be linked to the bloom-forming potential of the planktonic cyanobacterium *Planktothricoides* sp., which has more metabolic activity with metabolic partitioning for both growth and the production of secondary metabolites. These broad groups of secondary metabolites have beneficial uses, such as in pharmaceutical and industrial compounds, as well as in the biodegradation and production of vitamins (Figure 2). In addition, different light intensities had a major impact on the photosynthetic efficiency of *Planktothricoides* sp., which was related to their habitat. These species possess gas vesicles, and these vesicles are more abundant when the light intensity is reduced. Hence, at a low light level, the growth rate became slow and led to a lower production of secondary metabolites. This buoyancy regulation has a lot of advantages in comparison with other phytoplankton species.

### 3.2. Effects of Different Temperatures on the Growth and Metabolic Profiling of Hapalosiphon sp. MBR220 and Planktothricoides sp. SR001

In our analysis, the growth rate of *Hapalosiphon* sp. was found to be the highest at 33 °C, similar to what we found for *Planktothricoides* sp. (Table 2). Experimental evidence showed a positive correlation between cyanobacterial biomass levels and an increase in water temperatures [63,64]. It also was shown that the upper thermal limit (UTL) for the growth of different cyanobacteria in laboratory conditions was species-dependent, and varies from ~30 to 35 °C; in many species, the UTL is <33 °C [65]. However, the mechanism of this effect of temperature on increasing the algal bloom and off-flavor production is not known yet. This could be related to their habitat or adaptation to a changing environment, or a combination of both. Hence, a systematic understanding of the potential effects of both light and temperature through metabolite profiling would provide some insight into the metabolic activities of these cyanobacteria.

The metabolic profiling of *Hapalosiphon* sp. suggested that at varied temperatures, they produced both primary and secondary metabolites (Figure 3). Many of them played an important part in the defense mechanism to survive in complex environments, and provided resources to increase their competences regarding bloom development. This coincided with the information that an increase in temperature from mid to late summer caused massive blooms of a benthic cyanobacteria belonging to the genus *Ostreopsis*, which is harmful to human health in some urbanized temperate areas [65]. Temperature demonstrated a more profound effect than light intensity, with a higher number of amino acids involved in the biosynthesis/metabolism. The metabolism of phenylalanine, a precursor of secondary metabolites such as antitoxins, alkaloids, lignin, and flavonoids, in response to different temperature suggested that these metabolites might play a key role in regulating tolerance to temperature/heat stress. Hence, temperature could influence the expression of P450 enzymes in the thylakoid to participate in the phenylpropanoid biosynthesis. However, some of the metabolites associated with phenylpropanoid biosynthesis pathway could not be verified due to a lack of information in the cyanobacterial databases. The identification of tyrosine metabolism can be linked to aspartate metabolism and the synthesis of hormonelike molecules, as well as flagellar assembly (quorum sensing). It is also an important node in the cAMP signaling pathway. However, the presence of flagella is not known in cyanobacteria, yet they depend on the T4P for motility, a protein filament that extends from a membrane-spanning pore complex for mobility. This allows them to move toward a favorable environment from an unfavorable stress condition, such as intensity of light/high temperature. The cAMP signaling pathway is important, as it plays a key role in the regulation of various biological activities by controlling gene expression levels in respiration, light sensing, cell motility [66], and sensing with carbon acquisition [67]. In cyanobacteria, cAMP represents a high carbon signal [68]. During abiotic stress conditions, this signaling pathway plays a major role in the adaptation to the stress condition [69]. Interestingly, we identified a metabolite that was linked to lysine degradation, and this degradation could probably fuel this pathway for the biosynthesis of antibiotics, which was not observed in the response to different light intensities of this species. Pathways linked to antibiotics such as streptomycin were also observed in *Hapalosiphon* sp. in response to the temperature effect.

Surprisingly, different temperature levels had some pronounced effects on the metabolic activities of *Planktothricoides* sp. (Figure 4). Only a few metabolites linked to the biosynthesis of arginine and the metabolism of cysteine and methionine were identified. Arginine is a major storage and transport form of amino acid, the precursor for polyamines and nitric oxide (NO), and an essential metabolite for cellular and developmental processes. This amino acid can act as a signaling molecule that regulates essential cellular functions such as protein synthesis, apoptosis, and growth during critical conditions. It could also be acting as a twin-arginine translocation (Tat) pathway involved in the transport of proteins into and across the plasma and thylakoid membranes [70]. In cyanobacteria, the plasma and thylakoid membranes carry out very different activities, and arginine could play a key role in the transport of proteins. The metabolism of methionine is necessary for growth and development, as it acts as an initiator of protein synthesis (Figure 4). In addition, it functions as an endogenous antioxidant and redox sensor during unfavorable conditions [71,72]. It also increases the survival rate by modulating autophagy, or by inducing mitochondrial function and antioxidant defense [73]. Likewise, cysteine inhibits several enzymes of amino acid synthesis; therefore, increasing cysteine concentrations could increase the levels of the inhibited enzymes. It acts as a limiting factor for glutathione biosynthesis, which can be especially crucial for cyanobacteria, which rely on both the sufficient sulfur supply from the growth media and on the protection of glutathione against ROS that are produced during photosynthesis. Hence, it has the power to protect against oxidative stress. Although the metabolic activities were restricted at certain temperatures, this species still had the potential to degrade aromatic compounds such as naphthalene, benzene, and xylene to provide substrates for growth. Even though the effects of high temperature restricted the growth and metabolic activities of *Planktothricoides* sp., they clearly suggested the mechanism of adaptation to higher temperatures by increasing the survival rate through protection and antioxidant defense. This behavior was related to the habitat of this species, as they normally grow in local reservoirs in Singapore where the temperature varies between 28 and 34° C. This cyanobacterium can sustain many types of chemical stresses due to its capability for metabolic adaptations, and can also be used to clean up ccontaminated environments. The metabolite analysis also coincided with the observation that the growth of *Planktothricoides* sp. declined at the highest temperature; i.e., 38 °C.

The overall results suggested that different temperatures had more insightful effects on the metabolome of *Hapalosiphon* sp. compared to the effects of different light intensities. They survived higher light conditions through primary energy-producing metabolic pathways and amino acid metabolism. In contrast, the response of *Planktothricoides* sp. to different light intensities and temperatures was opposite to that of the *Hapalosiphon* sp., which could be related to their habitats, potentials for bloom formation, and structural differences. The light intensity had a more profound effect on the metabolome of this planktonic species compared to the effects of temperature. This suggested that the planktonic species has a higher tolerance capacity for high light intensities through carotenoid production, which protects the cells from photoprotection. The stress-responsive amino acid proline in *Planktothricoides* sp. could possibly play a role in scavenging reactive oxygen species (ROS) by stimulating an antioxidant defense mechanism in response to high light intensities. Similarly, ascorbate metabolism could be protecting the cyanobacterial cells from damage by ROS. The metabolism of methane suggests an alternative carbon source for their growth and metabolism [74]. Interestingly, they have the ability to sustain different types of chemical stresses through metabolic adaptation by degrading a number of aromatic compounds in response to high light intensities. Moreover, they showed the abilities to cope with imbalances in response to higher-temperature conditions through the accumulation of nitrogen storage compounds, acclimatize to higher temperature conditions through different survival and protection mechanisms, and degrade industrial pollutants. This is the first report on the metabolic profiling of two diverse cyanobacteria from different habitats in response to environmental changes. The information gathered from our analysis can be the basis for understanding the alterations in the metabolic activities of these two taxa for physiological and environmental adaptations. In addition, it also can assist in guiding biotechnological applications and strain designs. A more detailed analysis of endogenous and exogenous metabolites and their localizations for specific classes/groups will help in deciphering the variations in enzyme level and gene expression patterns associated with cellular responses to environmental stimuli and their combinations. This work on metabolic profiling suggested that a number of targeted metabolomics could be conducted to quantify metabolic changes under such environmental factors/stressors that would elucidate dominating metabolic pathways more precisely. There are also numerous toxic cyanopeptides produced by cyanobacteria besides the well-known microcystins [75,76]. Recent developments in advanced analytical techniques have facilitated the identification of those cyanopeptides. In the future, it would be interesting to identify the effects of light and temperature on different cyanopeptides. Additionally, a comprehensive understanding of the impact of environmental factors on cyanobacterial characteristics and an evaluation of the risk of cyanotoxin generation can help in managing the bloom control in and quality of water.

## 4. Materials and Methods

### 4.1. Effects of Light Intensity and Temperature

*Hapalosiphon* sp. and *Planktothricoides* sp. were respectively isolated from a sediment sample of an urban freshwater water body and a freshwater reservoir, both located in Singapore. They were inoculated in culture flasks containing MLA medium [77] and incubated in a plant growth chamber (Percival Scientific, IA, USA) at a constant temperature of 28 °C. The cultures were treated at different levels of light intensity, ranging from low to high (10 ± 3, 25 ± 3, 50 ± 3, and 100 ± 3 µmol photons m^−2^s^−1^) under different temperature levels (25, 28, 33, and 38 °C). The growth rates of the isolates under various conditions were calculated. All treatments were performed in three replicates.

The growths of the isolates were determined in terms of optical density (OD) at 750 nm at different treatment days (16, 20, and 24 days) using a UV–vis spectrophotometer (BioSpectrophotometer, Eppendorf, Germany). Linear correlations were obtained between the OD750 and cell abundance in each experiment (R^2^ = 0.83–0.85). The results of the biovolume for cell abundance and the OD measurements of *Planktothricoides* sp. are provided in Supplementary Materials S3. The slope of the growth curve [optical density vs. time (day)] was used as the growth rate.

### 4.2. Profiling of Metabolites

A total of 5 mL of cyanobacterial culture was harvested at each time point and filtered through a 0.22 µm Sartorius Minisart syringe filter (Göttingen, Germany) using a membrane holder/syringe assembly. The filter was then washed twice with cold water (4 °C) to remove excess media and salts. The filter was then carefully recovered from the membrane holder and transferred into a 15 mL conical centrifuge tube. It was subsequently snap-frozen in liquid nitrogen to quench the metabolic activity and stop any on-going biochemical reactions. At each time interval, the culture medium without algae (as the sample matrix) was also collected in the same way to eliminate the background signals. Samples were kept at −80 °C prior to analysis.

For the metabolic profiling, 80% methanol (*v*/*v*) in water was used as the extraction solvent. Briefly, 80% methanol precooled to −20 °C was added to the centrifuge tubes containing the filter. The volume of the solvent was adjusted accordingly to ensure the full immersion of the filter. The mixture was then bath-sonicated at full power for 20 min in a water bath at 4 °C to avoid heat generation. After sonication, the extract was separated by 0.22 µm syringe filters into a fresh 15 mL centrifuge tube, and was diluted with ultrapure water to 20% methanol and frozen at −80 °C before lyophilization at −105 °C. The dried sample was then reconstituted with 100 µL of 50% (*v*/*v*) methanol prior to analysis.

All chromatographic separations were carried out on a Thermo Accela 1250 Quaternary LC system, using a Kinetex XB-C18 (2.1 × 100 mm, 1.7 micron) reversed-phase column (Phenomenex, Torrance, CA, USA). The column and auto-sampler temperature were set to 45 and 6 °C, respectively. The mobile phase used was ultrapure water with 0.1% formic acid (as solvent A) and LC-MS grade acetonitrile with 0.1% formic acid (as solvent B) with the following gradient elution program: 2% B kept for 1 min, followed by an 11 min linear gradient to 98% B, which was kept until 13 min, then returned to 2% B at 13.5 min, followed by 4.5 min of column washing. The flow rate was set to 0.3 mL/min, and the injection volume was 10 µL.

An LTQ Orbitrap Velos Pro mass spectrometer (Thermo Scientific, Waltham, MA, USA) was used as the high-resolution mass spectrometer (MS) for the profiling. The analyses were carried out in a mass range of 60–1200 *m*/*z* at a resolution of 60,000, with a source voltage of 4 kV. The capillary and source heater temperatures were maintained at 300 °C with sheath gas, auxiliary gas, and sweep gas flow rates of 45, 15, and 1 (arbitrary units), respectively. The Xcalibur v2.2 software package (Thermo Scientific Inc., Waltham, MA, USA) was used for acquisition and preliminary raw data processing.

### 4.3. Data Analysis

Raw MS data were converted to the mzXML format using open-source Proteowizard software, then preprocessed using the open-source R-based XCMS software Version 3.2 for molecular feature detection and alignment. All data were then normalized by sample bio-volume and subjected to log transformation and quantile normalization. SIMCA-P Ver. 14 software (Umetrics AB, Umeå, Sweden) was used for the statistical analysis. Statistically significant features were analyzed using PerMANOVA. Differential features (with *p*-value < 0.05) were detected using permutation F-tests (N = 1000 permutations) in R.

### 4.4. Metabolic Pathway Mapping

Differential mass features identified in various data sets were mapped onto the metabolic network of the model cyanobacteria, *Microcystis aeruginosa* in the KEGG pathway (https://www.genome.jp/kegg-bin/show_organism?menu_type=pathway_maps&org=mar) (accessed on 26 January 2021). The putative identities of the key metabolites and their corresponding metabolic pathways perturbed in *Planktothricoides* sp. and *Hapalosiphon* sp. under various light intensity and temperature levels were determined during various growth stages.

## Figures and Tables

**Table 1:** Growth rates under different light intensity conditions.

Light Intensity (µmol Photons m^−2^s^−1^)	Growth Rate (Day^−1^)	
	*Hapalosiphon* sp. (MRB 220)	*Planktothricoides* sp. (SR001)
10 ± 3	0.067 ± 0.004	0.090 ± 0.008
25 ± 3	0.055 ± 0.005	0.200 ± 0.021
50 ± 3	0.068 ± 0.005	0.260 ± 0.025
100 ± 3	0.073 ± 0.007	0.320 ± 0.038

**Table 2:** Growth rates under different temperature conditions.

Temperature (^°^C)	Growth Rate (Day^−1^)	
	*Hapalosiphon* sp. (MRB 220)	*Planktothricoides* sp. (SR001)
25	0.064 ± 0.005	0.100 ± 0.009
28	0.057 ± 0.006	0.120 ± 0.014
33	0.070 ± 0.009	0.160 ± 0.018
38	0.060 ± 0.006	0.130 ± 0.014

**Table 3:** Effects of light and time on the metabolites in *Hapalosiphon* sp. (MRB 220))—PerMANOVA.

Factor	Df	Sums of Squares	Mean of Squares	F-Value	R^2^
Time point	2.00	0.29	0.15	10.50	0.17 *
Light	3.00	0.34	0.11	8.17	0.19 *
Time point * Light	6.00	0.33	0.05	3.93	0.18 *
Residuals	58.00	0.81	0.01		0.46 *
Total	69.00	1.77	1.00		

Df: degree of freedom; * significant effects on metabolite profiling based on PerMANOVA.

**Table 4:** Effects of light and time on the metabolites in *Planktothricoides* sp. (SR001)—PerMANOVA.

Factor	Df	Sums Of Squares	Mean of Squares	F-Value	R^2^
Time point	1.00	0.001	0.001	2.526	0.069 *
Light	3.00	0.003	0.001	2.952	0.24 *
Residuals	25.00	0.009	0.000	0.687	0.68 *
Total	29.00	0.014		1.000	

Df: degree of freedom; * significant effects on metabolite profiling based on PerMANOVA.

**Table 5:** Effects of temperature and time on the metabolites in *Hapalosiphon* sp. (MRB 220))—PerMANOVA.

Factor	Df	Sums Of Squares	Mean of Squares	F-Value	R^2^
Time point	2.00	0.06	0.03	14.09	0.19 *
Temperature	3.00	0.08	0.03	14.01	0.28 *
Time point * Temperature	6.00	0.04	0.01	3.26	0.13 *
Residuals	60.00	0.12	0.00		0.40
Total	71.00	0.30	1.00		

Df: degree of freedom; * significant effects on metabolite profiling based on PerMANOVA.

**Table 6:** Effects of temperature and time on the metabolites in *Planktothricoides* sp. (SR001)—PerMANOVA.

Factor	Df	Sums Of Squares	Mean of Squares	F-Value	R^2^
Time point	3	0.050999	0.0169997	15.5196	0.3 *
Temperature	3	0.012636	0.0042119	3.8452	0.07 *
Time point * Temperature	9	0.022259	0.0024732	2.2579	0.13 *
Residuals	78	0.085439	0.0010954		0.5 *
Total	93	0.171332	1		

Df: degree of freedom; * significant effects on metabolite profiling based on PerMANOVA.

## Data Availability

Data is contained within the article or supplementary material.

## Supplementary Materials

The following are available online at https://www.mdpi.com/article/10.3390/metabo12050406/s1, Supplementary Materials S1: a list of differential metabolites identified for *Hapalosiphon* sp. (MRB 220) (after mapping onto the KEGG metabolic pathway of *Microcystis aeruginosa*) in response to light and temperature, Supplementary Materials S2: a list of differential metabolites identified for *Planktothricoides* sp. (SR001) (after mapping onto the KEGG metabolic pathway of *Microcystis aeruginosa*) in response to light and temperature. Supplementary Materials S3: the results of biovolume for cell abundance and optical density measurements of *Planktothricoides* sp.
